# Clinical Factors Associated with Anxiety Disorders in Adults with Childhood Hypospadias Repair: Results from the F.R.E.S.H. Study

**DOI:** 10.3390/jpm16090472

**Published:** 2026-09-15

**Authors:** Michele Gnech, Massimiliano Buoli, Federica Fanti, Giulia Rotondi, Federica Marceca, Francesca Mitzman, Fabio Ciamarra, Giulia Garelli, Dario Guido Minoli, Erika Adalgisa De Marco, Daniele Stroppa, Massimo Di Grazia, Camilla Taverna, Eduje Thomas, Marco Castagnetti, Gianantonio Manzoni, Alfredo Berrettini

**Affiliations:** 1 Department of Paediatric Urology, Fondazione IRCCS Ca’ Granda Ospedale Maggiore Policlinico, 20122 Milan, Italy; federica.fanti97@gmail.com (F.F.); giulia.rotondi@policlinico.mi.it (G.R.);; 2 Department of Neurosciences and Mental Health, Fondazione IRCCS Ca’ Granda Ospedale Maggiore Policlinico, 20122 Milan, Italy; 3 Department of Pathophysiology and Transplantation, University of Milan, 20122 Milan, Italy; 4 Paediatric Surgery Department, IRCCS Istituto Giannina Gaslini, 16147 Genoa, Italy; 5 Department of Neuroscience, Rehabilitation, Ophthalmology, Genetics, Maternal and Child Health (DINOGMI), University of Genoa, 16132 Genoa, Italy; 6 Ausl Romagna, Ospedale Infermi, 47923 Rimini, Italy; 7 Pediatric Urology Unit, Bambino Gesù Children Hospital and Research Center, 00165 Rome, Italy

**Keywords:** hypospadias, urethroplasty, anxiety, long-term outcomes, depression, erectile dysfunction, penile perception, surgical complications, psychological assessment, sexual health

## Abstract

**Objectives:** This study aimed to evaluate the prevalence of pathological anxiety and to identify clinical, functional, and surgical factors associated with anxiety disorders in adults who underwent hypospadias repair during childhood. **Patients and Methods:** This cross-sectional analysis included adult males (≥18 years) previously treated surgically for hypospadias in childhood who underwent clinical evaluation and completed validated questionnaires assessing anxiety (State–Trait Anxiety Inventory, STAI-Y1/Y2), depression (Beck Depression Inventory-II), urological function (uroflowmetry and International Prostate Symptom Score), sexual function, and genital perception. Pathological anxiety was defined as STAI-Y1 ≥ 40. Patients with and without anxiety were compared using univariate analyses, and variables reaching statistical significance were entered into a binary logistic regression model to identify clinical variables independently associated with anxiety. **Results:** A total of 302 patients who underwent hypospadias surgery between 1975 and 2005 were included in this study. Of the 302 traced patients, 113 (37.4%) were contacted successfully, and a total of 36 adult male patients met the inclusion criteria for this study (mean age at assessment 23.6 years). Pathological anxiety was present in 14 patients (38.9%). Compared with non-anxious individuals, anxious patients were younger at assessment (*p* = 0.012), showed more impaired voiding dynamics, with lower flow index values (*p* = 0.060), and reported more severe urinary symptoms (*p* = 0.050). Depressive symptoms were significantly higher in the anxiety group (BDI-II: 9.5 vs. 3.1; *p* < 0.001) and were independently associated with pathological anxiety on multivariable analysis (OR 1.84, 95% CI 1.16–2.92; *p* = 0.01). Postoperative complications were more frequent and more severe among anxious patients, although this did not reach statistical significance. Subjective and objective aesthetic outcomes did not differ between groups. **Conclusions:** This study demonstrates that anxiety in adults treated for hypospadias during childhood is more closely related to functional urinary impairment, surgical complications, and depressive symptoms than to aesthetic outcomes. These findings emphasise the need for long-term, multidisciplinary follow-up incorporating routine mental health assessment. Early psychological screening and targeted supportive interventions may be associated with better functional and psychosocial outcomes, supporting a holistic, integrated model of care for hypospadias patients.

## 1. Introduction

Hypospadias is a congenital penile anomaly characterised by the abnormal positioning of the external urethral meatus, which may be located anywhere along the ventral surface of the penile shaft or, more rarely, in the scrotum or perineum. This condition is also commonly associated with a ventral preputial defect and varying degrees of penile curvature. Hypospadias is considered a multifactorial disorder, arising from the interplay of genetic and environmental factors, with significant variation in presentation among individuals [1]. The heterogeneity in this defect has necessitated the development of numerous surgical techniques for primary repair over the years. The primary goals of surgical intervention are to achieve both functional and aesthetic reconstruction, which includes ensuring a straight penile shaft and positioning the external urethral meatus at the tip of the glans [2].

This manuscript is the first from the F.R.E.S.H. study (Functional, Reconstructive, Esthetic, Social, and Psychological outcomes in Hypospadias patients treated in childhood), involving adult males treated by a single paediatric urologist. Participants completed validated questionnaires on quality of life, sexual orientation, and psychological outcomes and underwent medical examinations of aesthetic and functional results. A detailed protocol of the study is provided in Appendix A.

While prior studies focused on the functional and aesthetic outcomes of hypospadias repair, the long-term psychiatric impact of this genital malformation and its surgical treatment, particularly in adulthood, remains largely unexplored. Of note, anxiety disorders represent prevalent conditions associated with social dysfunction and poor quality of life [3]. The presence of these disorders should be identified in subjects operated on for hypospadias because it could compromise adherence to medical prescriptions and perceived quality of surgery, as well as worsening the patients’ well-being [4,5,6]. In addition, anxiety disorders are frequently in comorbidity with depression, further complicating the course of patients affected by hypospadias [7,8].

The primary aim of this manuscript is to explore the prevalence and severity of pathological anxiety within this specific population. The secondary objective is to identify potential clinical and surgical factors in the medical and surgical histories of these patients that may predispose them to anxiety disorders.

## 2. Materials and Methods

Ethical approval was obtained, and all participants provided written informed consent. Data were collected retrospectively from patient medical records and prospectively through patient interviews. All consecutive adult male patients aged 18 years or older who had undergone hypospadias surgery before the age of 18 were included in the study.

The statistical analysis aimed to investigate the prevalence of anxiety in this particular population and to identify any predisposing factors in patients’ clinical histories based on the collected variables. For this analysis, patients who had completed the State–Trait Anxiety Inventory (STAI-Y1 and STAI-Y2) but had not undergone a clinical visit were excluded [9].

A complete-case analysis was adopted. For this anxiety-focused analysis, only patients with complete STAI-Y1 data were included. Among 56 patients enrolled in F.R.E.S.H., 20 were excluded because the STAI-Y1/Y2 was not completed. No imputation was performed due to the small sample size.

### 2.1. Demographic and Clinical Variables

Collected variables included age at surgery and assessment, educational level, employment, marital/parental status, habitual medications, hypospadias phenotype, penile curvature, associated anomalies, additional procedures, and postoperative complications. To minimise recall bias, surgical history, hypospadias phenotype and early complications were primarily retrieved from original operative reports and hospital charts, while sociodemographic and functional data were collected prospectively.

### 2.2. Penile Outcomes

Penile appearance and patient satisfaction on surgery results were assessed using the Pediatric Penile Perception Score (PPPS) and the Satisfaction in Genital Hypospadias Treatment (SIGHT). [10,11] The Hypospadias Objective Scoring Evaluation (HOSE) was used to assess the aesthetic outcomes of the urethroplasty [12]. The final aesthetic result was ultimately evaluated by the urologist in charge of the visit using the Hypospadias Objective Penile Evaluation (HOPE) scale to be as objective as possible [13].

### 2.3. Urological Functional Outcomes

To evaluate long-term urological function, patients underwent uroflowmetry testing and post-void residual volume (PVR) measurement via ultrasound. Key uroflowmetry parameters, such as voided volume (VV), maximum urinary flow rate (Qmax), and average urinary flow rate (Qave), were recorded. The flow index (FI) was employed to classify the uroflow curves [14]. The International Prostate Symptom Score (IPSS) was utilised as a validated test to support the objectification of urinary symptoms, even though it is not specific to this population [15].

### 2.4. Sexual Orientation and Gender Identity

In the study, participants provided information on their declared sexual orientation and perceived gender. Additionally, they completed two validated questionnaires: the Klein Sexual Orientation Grid (KSOG) and the Gender Identity/Gender Dysphoria Questionnaire for Adolescents and Adults (GIDYQ-AA).

The KSOG was developed to assess various aspects of sexual orientation, including sexual behaviour, attraction, sexual fantasies, social preferences, emotional preferences, self-identification, and lifestyle. These factors are evaluated across three time dimensions: the past, the present, and the ideal.

The GIDYQ-AA is a 27-item self-report measure designed to assess various dimensions of gender identity and dysphoria. The GIDYQ-AA evaluates subjective experiences, physical aspects, social interactions, and socio-legal considerations related to gender dysphoria. The questionnaire has demonstrated strong discriminant validity, effectively distinguishing individuals with gender dysphoria from control groups [16,17].

### 2.5. Sexual Function Assessments

Sexual function was evaluated using the International Index of Erectile Function (IIEF-15) [18].

### 2.6. Mental Health Assessment

Mental health assessment was performed using a range of validated questionnaires. Anxiety symptoms were evaluated through the State Anxiety Inventory (STAI), a self-report questionnaire that measures the presence and severity of current symptoms of anxiety (STAI-Y1) and a generalised propensity to be anxious (STAI-Y2) [9]. A STAI-Y1 score ≥ 40 is indicative of pathological anxiety [19]. Depression was evaluated using the Beck Depression Inventory-II (BDI-II), while suicidal tendencies were assessed with the Multi-Attitude Suicide Tendency Scale (MAST). A BDI-II total score of 0–13 is considered minimal, 14–19 is mild, 20–28 is moderate, and 29–63 is severe [20,21].

### 2.7. Statistical Analysis

Data were analysed using IBM SPSS Statistics Base (v.29.0). Descriptive analyses were performed. The two groups identified by the presence of anxiety disorders (STAI-Y1< or ≥40) were compared by unpaired sample *t* tests for continuous variables and χ^2^ tests (with odds ratio—OR—calculation where appropriate) for qualitative variables. Statistically significant variables in the univariate analyses were entered as independent factors in a binary logistic regression model with presence of pathological anxiety (STAY-Y1 ≥ 40) as the dependent variable to identify factors associated with anxiety. The reliability of the model was assessed by the Omnibus and Hosmer–Lemeshow tests. Finally, correlation analyses were performed between STAI-Y1 scores and continuous variables.

## 3. Results

### 3.1. Demographic and Clinical Variables

**Figure 1:**
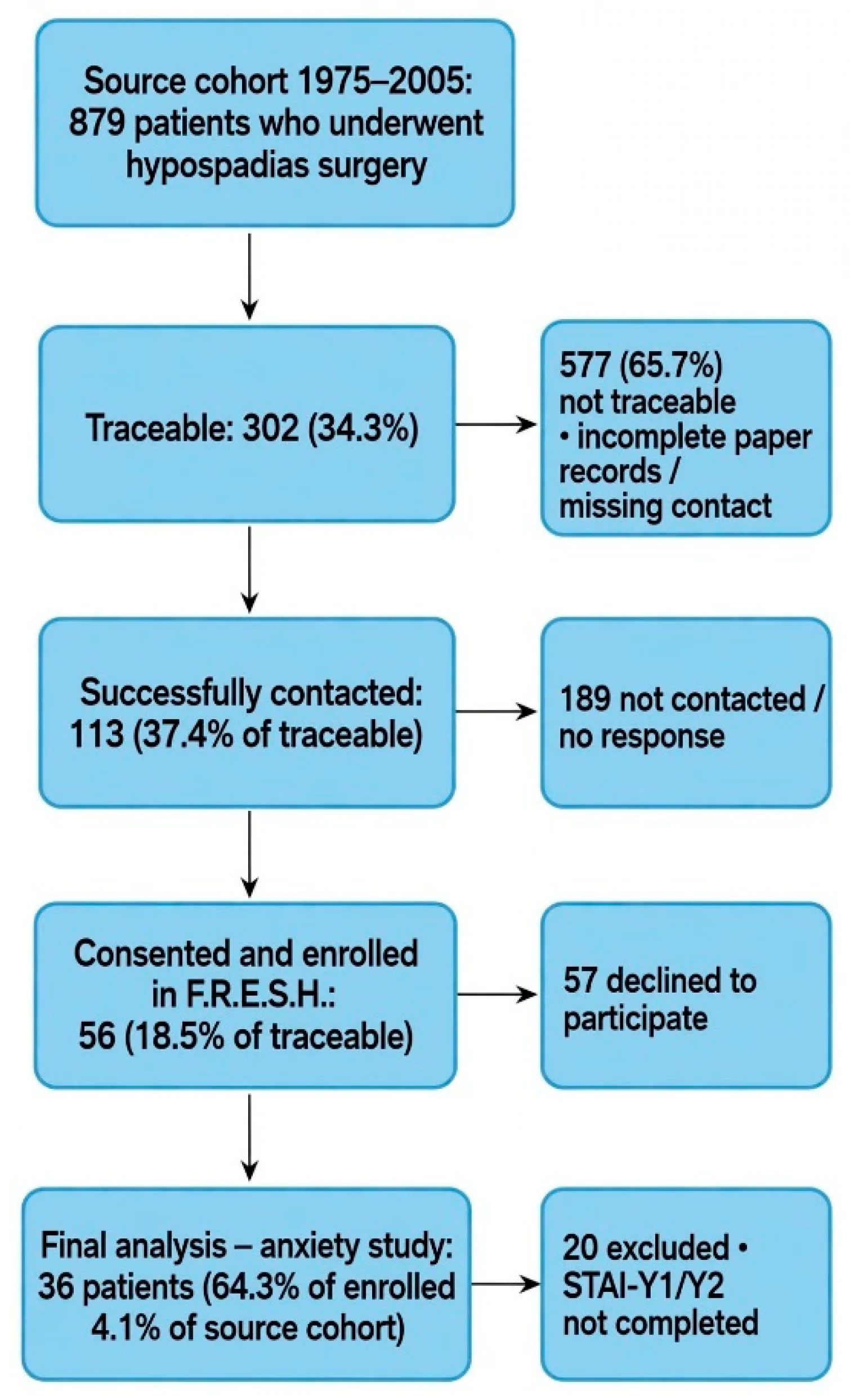
STROBE flow diagram of study participants.

Of 879 patients operated on between 1975 and 2005, 577 (65.7%) were not traceable due to incomplete paper archives. Of 302 traceable patients (34.3%), 113 (37.4%) were successfully contacted. Fifty-seven declined to participate in the F.R.E.S.H. study. Fifty-six were enrolled. Twenty of these patients did not complete the STAI-Y1/Y2 and were excluded from this anxiety analysis. The final analysis includes 36 patients (64.3% of enrolled; 4.1% of the source cohort). Participant flow is shown in Figure 1 (STROBE).

The mean interval between first urethroplasty and clinical assessment was 19.8 ± 7.2 years, with a wide range (from 13 to 43 years), reflecting the 30-year inclusion period (1975–2005).

Regarding hypospadias phenotypes, the majority of patients had distal hypospadias (21 patients, 58.3%), followed by penile hypospadias (11 patients, 30.6%) and proximal hypospadias (three patients, 8.7%). One patient (2.4%) had an unknown classification. Associated penile curvature was identified in 10 patients (27.8%), and 13 patients (36.1%) presented with associated anomalies, such as cryptorchidism, which required coordinated surgical management in 10 cases (27.8%) (Table 1).

### 3.2. Sociodemographic and Socioeconomic Characteristics

The cohort exhibited a heterogeneous socioeconomic background; 11 patients (30.5%) had attained a university degree. Thirteen participants (36.1%) were married, and three (8.3%) had children (Table 1).

**Table 1:** Demographic and clinical variables and sociodemographic and socioeconomic characteristics.

**Cohort Selection**	**Value (%)**
Total cohort size (1975–2005)	879
Total number of patients included in the F.R.E.S.H. study	302 (34.3%)
Patients not traced (due to incomplete contact/loss to follow-up)	577 (65.7%)
Patients contacted successfully	113 (37.4%)
Patients who agreed to participate in the F.R.E.S.H. study	56 (18.5%)
Total number of adult male patients for the study on anxiety	36
**Clinical Variables**	**Value (%)**
Mean age at the time of surgery	45.85 ± 48.84 months
Mean age at the time of study visit	23.58 ± 6.61 years
Hypospadias Phenotype at Birth	
- Distal	21 (58.3)
- Penile	11 (30.6)
- Proximal	3 (8.7)
- Unknown	1 (2.4)
Associated Penile Curvature	10 (27.8)
Patients with Associated Anomalies	13 (36.1)
Surgical Management for Anomalies	10 (27.8)
**Sociodemographic Characteristic**	**Value (%)**
Educational Attainment	
- 18 years of education	7 (19.4)
- 16 years of education	4 (11.1)
- 13 years of education	18 (50.0)
- 8 years of education	6 (16.7)
- 5 years of education	1 (2.8)
Employment Status	34 (94.4)
Married	13 (36.1)
Have Children	3 (8.3)
Regular Medication Use	
No habitual medication	27 (75.0)

### 3.3. Complications

Complications following hypospadias surgery were reported in 24 patients (66.7%). Specifically, 18 patients (50.0%) experienced a fistula, five patients (13.9%) had urethral stenosis, and one patient (2.8%) developed meatal stenosis. The severity of the major complication was stratified according to the Clavien–Dindo classification. The average number of complications per patient was two (range: 1–4).

Among the patients with a history of complications, four individuals (11.1%) still had a persistent fistula at the time of the study visit. Residual penile curvature was observed in nine patients (25.0%) (Table 2).

### 3.4. Penile Outcomes

The PPPS was available for 36 patients, with a minimum score of 0 and a maximum score of 12, indicating full satisfaction with the perceived aesthetic outcome. The mean score in our cohort was 7.97 ± 2.29.

The SIGHT questionnaire revealed an average score of 26.17 ± 2.60, with satisfaction levels ranging from 11 to 45. Notably, responses to question 10, regarding the age of first sexual intercourse, showed a mean of 17.9 ± 3.2 years.

The HOSE questionnaire classified outcomes for 35 patients, with scores ranging from 5 to 16, where 16 represents an optimal result. The mean HOSE score was 11.68 ± 1.22.

An objective assessment of the aesthetic outcome was performed using the HOPE scale. The mean value for the population was 42.85 ± 5.82, with a range varying from a minimum of 5 to a maximum of 50 (Table 2).

### 3.5. Urological Functional Outcomes

Urological function was assessed using uroflowmetry and the IPSS questionnaire, showing overall preserved urinary flow parameters with a substantial proportion of patients reporting mild-to-moderate lower urinary tract symptoms. Flow curve types observed included five patients (13.9%) with tower-shaped curves, 15 patients (41.7%) with bell-shaped curves, and 16 patients (44.4%) with plateau-shaped curves.

Urinary symptoms, as measured by the IPSS, indicated mild symptoms in 22 patients (61.1%) and moderate symptoms in 12 patients (33.3%), while two patients (5.6%) did not complete the IPSS questionnaire (Table 2).

### 3.6. Sexual Orientation and Gender Identity

All participants self-reported identifying as male, and two individuals (5.6%) reported themselves as homosexual. According to the KSOG, 13 were prevalently heterosexual, two prevalently homosexual and three bisexual. The mean GIDYQ-AA score was 4.55 (1 = non-gender conformity; 5 = total gender conformity).

### 3.7. Sexual Function Assessments

Of the 36 participants, 18 (50%) completed the IIEF-15 questionnaire but reported no sexual activity in the preceding four weeks. Twelve patients (66.7%) had no erectile dysfunction, four patients (22.3%) had mild erectile dysfunction, one patient (5.5%) had moderate erectile dysfunction, and one patient (5.5%) had severe erectile dysfunction. Among those sexually active, only two patients (11.1%) reported unsatisfactory sexual intercourse. In relation to orgasmic function, seven patients (19.5%) reported reduced ability to achieve orgasm. A low sexual desire was reported by 10 patients (27.8%). Finally, overall satisfaction was assessed, with 17 patients (47.2%) indicating they were satisfied with their sexual life (Table 2).

### 3.8. Mental Health Assessment

The STAI-Y1 identified 14 (38.9%) patients with pathological anxiety, of which 11 (30.6%) presented mild, two (5.6%) moderate, and one (2.8%) severe anxiety. The STAI-Y2 revealed 20 (55.6%) patients with a propensity for anxiety.

For the BDI-II, two patients (5.6%) were found to have pathological levels of depression. Of these, one (2.8%) exhibited mild depression, while one (2.8%) presented moderate depression.

According to the MAST, four patients (11.1%) exhibited a risk of self-harm.

### 3.9. Perioperative Factors Predisposing to Anxiety Disorders

The mean age at the time of the first urethroplasty was 43.54 ± 39.50 months in the anxiety group and 47.35 ± 55.47 months in the non-anxious group, with no statistically significant difference (*p* = 0.832). Similarly, the age at the first postoperative complication did not significantly differ between groups (8.55 ± 4.09 years vs. 8.90 ± 5.62 years; *p* = 0.118). At the time of clinical assessment, patients with anxiety were significantly younger than their non-anxious counterparts (21.00 ± 2.74 years vs. 25.23 ± 7.93 years; t = 2.29, *p* = 0.012). Hypospadias phenotype distribution was comparable (χ^2^ = 2.08, *p* = 0.698).

Sociodemographic factors such as educational attainment, employment status, and marital or parental status did not differ significantly between groups (*p* > 0.05). Employment rates remained high (>90%) in both cohorts.

Postoperative complications were more frequent in the anxiety group (85.8% vs. 54.5%), with a higher proportion of Clavien–Dindo grade 3 complications, although this difference did not reach statistical significance.

Aesthetic and patient satisfaction outcomes, as assessed using the PPPS and the SIGHT questionnaire, were marginally lower in the anxiety group. The mean PPPS score was 7.57 ± 1.95 in anxious patients compared to 8.27 ± 2.67 in non-anxious individuals. Similarly, SIGHT scores were slightly lower in the anxiety cohort (25.71 ± 2.5) versus the non-anxious group (26.45 ± 2.7). However, objective aesthetic evaluations using the HOPE scale did not reveal significant differences between groups (t = 0.53, *p* = 0.599).

The mean age at first sexual intercourse was comparable between groups (18.00 ± 2.5 years vs. 18.1 ± 3.7 years), with no statistically significant difference (t = 0.08, *p* = 0.939).

Urological function assessments, including uroflowmetry and symptom scores, demonstrated significant disparities between the two groups. The flow index (FI) was lower in the anxiety group (0.67 ± 0.24) compared to the non-anxious group (0.82 ± 0.29) with borderline statistical significance (t = 1.60, *p* = 0.060). The prevalence of plateau-shaped uroflow curves was higher in patients with anxiety, and it was the most common shape.

Urinary symptom severity, measured by the IPSS, tended to be higher in the anxiety cohort (mean: 7.69 ± 3.40) compared to the non-anxious cohort (mean: 5.48 ± 3.0; *p* = 0.050), with a borderline *p*-value (t = 1.99, *p* = 0.05). Moderate urinary symptoms were more prevalent among patients with pathological anxiety, whereas mild symptoms were prevalent in the non-anxiety group.

Erectile function, assessed using the IIEF-15, did not show a clear distinction between the groups. Among anxious patients, five out of 14 were sexually active, with one case of mild erectile dysfunction and one case of moderate erectile dysfunction. In non-anxious patients, 13 out of 22 were sexually active, with three cases of mild erectile dysfunction and one case of moderate erectile dysfunction.

Psychological assessments revealed a significant burden of depressive symptoms among patients with anxiety. Scores on the Beck Depression Inventory-II (BDI-II) were substantially higher in the anxiety cohort (9.50 ± 6.1) compared to the non-anxious group (3.14 ± 2.6) (t = 4.30; *p* < 0.001). Similar results were found for trait anxiety (STAI-Y2 scores) (t = 6.11, *p* < 0.001) (Table 3).

### 3.10. Binary Logistic Regression Analysis

The binary logistic regression model was reliable (Omnibus test: χ^2^ = 21.59, *p* < 0.001; Hosmer and Lemeshow test: χ^2^ = 5.22, *p* = 0.734), allowing for a correct classification of 79.4% of cases. Severity of depressive symptoms (BDI-II total scores) was found to be independently associated with the presence of anxiety (OR = 1.84, 95% CI 1.16–2.92; *p* = 0.01).

### 3.11. Correlation Analyses

These analyses confirmed a significant correlation between the STAI-Y1 and BDI-II total scores (Pearson’s r = 0.697, *p* < 0.001) and FI (r = −0.322, *p* = 0.05), as well as between the STAI-Y1 and IPSS at borderline statistical significance (r = 0.325, *p* = 0.06).

## 4. Discussion

The findings from the F.R.E.S.H. study provide novel insights into the psychiatric outcomes following childhood hypospadias repair, particularly concerning anxiety disorders in adulthood. This is a highly selected cohort (36/879, 4.1% of the source population, Figure 1); therefore, all rates are descriptive and not representative of the general hypospadias population.

### 4.1. Sociodemographic Factors

No significant differences were found between anxious and non-anxious patients regarding educational attainment, employment status, marital status, or parental status. Employment rates remained high (>90%) in both groups. This is consistent with population-based data showing equivalent social outcomes in men with hypospadias, including education, income and likelihood of marriage. No sociodemographic factor was associated with anxiety [22].

### 4.2. Hypospadias Phenotypes and Surgical Outcomes

In the F.R.E.S.H. cohort, the distribution of hypospadias types (distal: 50.0%, penile: 42.8%, proximal: 7.2% in the anxiety group) aligns with the existing literature, in which distal forms are the most common. No association was found between phenotype, age at first surgery or number of procedures and anxiety. This suggests that objective anatomical severity alone does not predict anxiety [23].

### 4.3. Postoperative Complications

Postoperative complications were more frequent in the anxiety group (85.8% vs. 54.5%, *p* = 0.076), with Clavien–Dindo Grade ≥3 in 35.8% vs. 22.9%. While not reaching statistical significance, this trend may suggest an association between a more complicated postoperative course and anxiety, to be confirmed in larger cohorts. Long-term complication rates after hypospadias repair range from 10% to 39% (fistula, meatal stenosis, voiding dysfunction), and up to 36% experience ≥1 complication, with 64% ≥ 2 complications in adult series. Functional impairments such as hesitancy and weak stream are reported in up to 39% [24,25,26,27]. 

In our cohort, the long heterogeneous follow-up (13–43 years) may have led to underestimation of early complications managed elsewhere, as acknowledged in the Limitations and Strengths Section.

### 4.4. Aesthetic and Satisfaction Outcomes

Despite higher complication rates, aesthetic satisfaction did not differ significantly. The PPPS was 7.57 ± 1.95 in anxious vs. 8.27 ± 2.67 in non-anxious patients; SIGHT was 25.71 ± 2.56 vs. 26.45 ± 2.70; and HOPE showed no differences. Gunawan et al. reported lower cosmetic satisfaction vs. controls, especially in proximal forms, without a direct link to psychological distress. Mureau et al. found ∼25% dissatisfaction with penile appearance (scars, size, glans shape) with similar rates in distal and proximal forms, but proximal cases reported more inhibition in seeking sexual contacts (50.0% vs. 18.9%, *p* = 0.033). Compared to Mureau (adolescents), our adult cohort shows slightly higher PPPS scores. Our data suggest that functional outcomes and psychiatric comorbidity may be more strongly associated with anxiety than aesthetic concerns alone [24,28,29].

### 4.5. Urological Function

Urological parameters tended to be more impaired in the anxiety group: FI 0.67 ± 0.24 vs. 0.82 ± 0.29 (*p* = 0.060) and IPSS 7.69 ± 3.40 vs. 5.48 ± 2.99 (*p* = 0.050). This borderline association suggests a possible link between perceived urinary dysfunction and anxiety, to be interpreted cautiously given the small sample size. Perera et al. reported lower Qmax vs. controls but within the normal range, while Bocchino et al. found a mean IPSS of 0.96 ± 1.97, indicating mild symptoms. González et al. noted that uroflowmetry may reveal subtle dysfunction not clinically apparent. Our higher IPSS, especially in anxious patients, suggests that perceived dysfunction may impact psychological distress [30,31,32].

### 4.6. Sexual Function Assessments

Sexual function was largely preserved. Bocchino et al. reported a mean IIEF-5 of 24.10 ± 1.02, comparable to our cohort. Mureau et al., using semi-structured interviews, found no differences in sexual behaviour vs. controls, but more negative genital appraisal and poorer sociosexual development when surgery occurred at an older age. Husmann reported ED in 63% of cases with multiple failed repairs, with plate division, ventral grafting and repeated urethrotomies as predictors. Our lower ED prevalence, and the association between fewer surgeries and preserved structures, underscores the negative impact of multiple procedures and supports a conservative approach [29,31,33].

### 4.7. Mental Health

The prevalence of pathological anxiety (STAI-Y1 ≥ 40) in our cohort was 38.9% (14/36), with 30.6% mild, 5.6% moderate and 2.8% severe anxiety. We did not have a matched healthy control; therefore, we referred to the published literature data for young males with a similar age range. This rate is substantially higher than estimates for the general population: the 12-month prevalence of anxiety disorders in young adult males in Europe is approximately 4–6% [34] and normative data for the Italian version of the STAI-Y1 indicate pathological scores (≥40) in approximately 15–20% of young men from the community [35]. It is also higher than the 21% prevalence of any psychiatric symptoms (including anxiety and depression) previously reported in adults with hypospadias by Örtqvist et al. While a directly matched healthy control group was not available in the present cross-sectional F.R.E.S.H. study, these comparisons suggest an increased burden of anxiety symptoms in adults operated on for hypospadias in childhood [34].

Unlike the mentioned cross-sectional study, which did not assess clinical or functional variables, our research establishes correlations between mental health outcomes and detailed clinical evaluations [34]. By incorporating physical examinations, we identified predisposing clinical factors associated with anxiety, offering a novel perspective on the interplay between clinical complications and the presence of anxiety disorders. Although our sample size is smaller, our study design provides greater accuracy in analysing these relationships. The original finding of the present study is that urinary symptoms rather than the appearance of the penis or sexual dysfunction were associated with the presence of anxiety disorders. This finding has been reported in several studies on patients with urinary disorders, but never in subjects with previous hypospadias, so voiding dysfunction should be taken into consideration for its impact on mental health [36].

As well as anxiety disorders, in our study, the BDI-II scores identified depressive disorders in two patients (5.6%): one (2.8%) with mild depression and one (2.8%) with moderate depression. Additionally, the MAST indicated that four patients (11.1%) were at risk of self-harm. Örtqvist et al. found that 21% of individuals with hypospadias experienced psychiatric symptoms, including anxiety and depression, though they did not quantify depression severity or self-harm risk [24]. Likewise, Gulseth et al. reported that 16% of adolescents who underwent hypospadias surgery were classified as individuals with full or sub-threshold psychiatric disorders compared to 3% in a control group [37]. These findings align with our results, which identified a 5.6% prevalence of clinical depression and an 11.1% risk of self-harm. In addition, anxiety disorders were found to be associated with the severity of depressive symptoms, indicating how these subjects may be vulnerable to various psychiatric conditions. The perspective of human sexuality involves multiple dimensions: biological, psychological, anthropological, social, relational, cultural, and interpersonal. These aspects are fundamental in understanding the sexuological needs of patients and allow for a comprehensive psychosexual assessment, including the evaluation of suitability for andrological surgery [38].

It is plausible that unmeasured psychosocial factors, including family functioning, perceived social support and partner support, may moderate the association between functional urological impairment and anxiety.

This underscores the necessity of integrating structured mental health screening and interventions into the long-term management of hypospadias patients, with the objective to limit the adverse effects of poor mental health.

## 5. Limitations and Strengths

This study has several limitations. First, the cross-sectional design precludes any inference of causality or temporal directionality between clinical variables and anxiety. For instance, we cannot determine whether depressive symptoms lead to anxiety or vice versa, or whether urinary symptoms increase anxiety or anxiety heightens symptom perception. Our results should therefore be interpreted strictly as associations. Additionally, the absence of a matched healthy control group limits the ability to quantify the excess risk specifically attributable to hypospadias compared to the general population; we therefore used published normative STAI-Y1 data as an external comparator only for historical contextualisation. A formal comparison with general population norms (is provided only for this purpose; these norms derive from a 1989 student/worker sample and do not represent a matched contemporary control group, and no claim of increased prevalence versus general population is made [35].

Second, attrition and non-response bias: participant flow is detailed in Figure 1 (STROBE). Of 879 source patients, 577 (65.7%) were not traceable due to incomplete paper archives, and a formal comparison of baseline characteristics between traceable and non-traceable patients was not possible for the same reason. Among 56 patients enrolled in F.R.E.S.H., 20 were excluded from this analysis due to missing STAI-Y1/Y2 data, representing further self-selection. Only 36/879 (4.1%) of the source cohort were analysed.

Third, the time span between primary hypospadias surgery (1975–2005) and adult assessment varied greatly (mean interval ∼20 years, range 13–43 years). This long and variable interval may affect both recall bias for early postoperative events and the long-term evolution of complications and urinary function, as a single cross-sectional evaluation cannot capture their dynamic course over decades. Moreover, the 30-year inclusion period implies substantial evolution in surgical techniques (MAGPI to TIP), suture materials, and perioperative care, which was not quantifiable from historical paper charts and could not be adjusted for in the analysis due to the small sample size. This heterogeneity represents an unmeasured confounder.

Finally, important psychosocial determinants of anxiety, such as perceived social support, family environment, parental attitudes during childhood, history of peer victimisation or bullying, and quality of partner/intimate relationship support, were not systematically assessed in the F.R.E.S.H. protocol. Although we recorded marital status, parental status and educational/employment status as proxies of social integration, these do not reflect the quality of support. Their absence may constitute unmeasured confounding, as low social support and adverse family environment are known to be independently associated with anxiety disorders.

Nonetheless, the study has notable strengths: it focuses on adult males with hypospadias, integrates validated psychiatric, functional, and aesthetic assessments, and combines physical and mental health parameters, providing a comprehensive understanding of surgical and psychosocial outcomes in this often-overlooked population.

## 6. Conclusions

This study highlights the intricate relationship between surgical history, functional impairments, and anxiety in adults who underwent childhood hypospadias repair. Our findings suggest that postoperative complications, impaired urinary function, and depressive symptoms may be associated with pathological anxiety, although these associations should be interpreted cautiously given the limited sample size. Due to the cross-sectional design, these associations do not imply causality and should be interpreted as correlations. Longitudinal studies are required to clarify temporal and causal relationships.

The identification of these potentially relevant clinical factors underscores the importance of a multidisciplinary follow-up approach that includes routine psychiatric screening alongside surgical monitoring. While previous research has predominantly focused on surgical and cosmetic outcomes, our study adds a critical psychosocial dimension, revealing that patients with hypospadias may benefit from early mental health interventions to prevent long-term distress.

Future research should prioritise longitudinal studies to better understand the evolving mental health trajectory of hypospadias patients over time. Additionally, the development of targeted interventions, such as structured counselling programs and tailored post-surgical support, may significantly enhance both functional and mental health outcomes in this population. By shifting the focus beyond purely surgical success, this study provides novel insights into the holistic management of hypospadias, reinforcing the necessity of an integrated care model that addresses both physical and mental well-being.

## Figures and Tables

**Table 2:** Complications, penile outcomes, urological functional outcomes and sexual outcomes.

**Complications**	**Value (%)**
Total Patients with Complications	24 (66.7)
Types of Complications	
- Fistulas	18 (50.0)
- Urethral stenosis	5 (13.9)
- Meatal stenosis	1 (2.8)
Severity of Complications (Clavien–Dindo Classification)	
- Grade 1	3 (8.3)
- Grade 2	11 (30.6)
- Grade 3	10 (27.8)
Average Number of Complications Per Patient	2 (1–4)
Persistent Fistula at Study Visit	4 (11.1)
Residual Penile Curvature	9 (25.0)
**Penile Outcomes**	**Value (%)**
PPPS (Penile Perception and Psychological Satisfaction)	
- Mean score	7.97 ± 2.29 (0–12)
SIGHT (Sexual Impact of Genital Surgery on Health and Well-Being)	
- Mean score	26.17 ± 2.60 (11–45)
- Age of first sexual intercourse (Q10)	17.9 ± 3.2 years
HOSE (Hypospadias Objective Scoring Evaluation)	
- Mean score	11.68 ± 1.22 (5–16)
HOPE (Objective Aesthetic Assessment)	
- Mean score	42.85 ± 5.82 (5–50)
**Functional Outcomes**	**Value (%)**
Uroflowmetry Results	
- Mean maximum urinary flow rate (Qmax)	19.63 ± 6.65 mL/s
- Mean average urinary flow rate (Qave)	10.26 ± 4.1 mL/s
- Mean voided volume (VV)	310.4 ± 163.2 mL
Median F.I.	0.734
Flow Curve Types (F.I.)	
- Tower-shaped curves (>1.253)	5 (13.9)
- Bell-shaped curves (>0.659 < 1.253)	15 (41.7)
- Plateau-shaped curves (<0.659)	16 (44.4)
Urinary Symptoms (IPSS)	
- Mild symptoms (0–7)	22 (61.1)
- Moderate symptoms (8–19)	12 (33.3)
- Severe symptoms (20–35)	0 (0)
- Did not complete the IPSS questionnaire	2 (5.6)
**Sexual Outcomes**	**Participants (%)**
Completion of IIEF-15 Questionnaire	36
- Completed but reported no sexual activity (previous 4 weeks)	18 (50.0)
Erectile Function (IIEF-15 Score)	18 (50.0)
- No erectile dysfunction (score: 26–30)	12 (66.7)
- Mild erectile dysfunction (score: 17–25)	4 (22.3)
- Moderate erectile dysfunction (score: 11–16)	1 (5.5)
- Severe erectile dysfunction (score: 0–10)	1 (5.5)
Sexual Activity	18 (50)
- Satisfactory sexual intercourse	16 (88.9)
- Unsatisfactory sexual experiences	2 (11.1)
Orgasmic Function	36 (100)
- Reduced ability to achieve orgasm (0–4)	7 (19.5)
- Normal ability to achieve orgasm (5–10)	29 (80.5)
Sexual Desire	36 (100)
- Low sexual desire (2–6)	10 (27.8)
- Normal sexual desire (7–10)	26 (72.2)
Overall Sexual Satisfaction	36 (100)
- Satisfied with sexual life	17 (47.2)
- Not satisfied with sexual life	19 (52.8)

**Table 3:** Perioperative factors predisposing to anxiety.

Clinical Variables	14 Patients with Anxiety Mean ± SD or (%)	22 Patients Without Anxiety Mean ± SD or (%)	*p*-Value
Age at the first:			
- Urethroplasty (months)	43.54 ± 39.50	47.35 ± 55.47	0.832
- Complication (years)	8.55 ± 4.09	8.90 ± 5.62	0.118
- Visit (months)	21.00 ± 2.74	25.23 ± 7.93	0.012
Hypospadias phenotype			0.698
- Distal hypospadias	7 (50.0)	14 (63.6)	
- Penile hypospadias	6 (42.8)	5 (22.5)	
- Proximal hypospadias	1 (7.29)	2 (9.3)	
- Unknown	0 (0)	1 (4.6)	
Associated penile curvature	4 (28.6)	6 (27.3)	0.891
Associated anomalies	4 (28.6)	9 (40.9)	0.591
Sociodemographic Variables			
Educational status			0.240
- 5 years	0 (0)	0 (0)	
- 8 years	1 (7.2)	4 (18.4)	
- 13 years	2 (14.2)	9 (40.3)	
- 16 years	9 (64.4)	4 (18.4)	
- ≥18 years	2 (14.2)	5 (22.9)	
Employment	13 (92.8)	21 (95.4)	0.743
Marital status (married)	3 (21.4)	10 (45.4)	0.175
Parental status (children)	0 (0)	3 (13.6)	0.267
Postoperative complications	12 (85.8)	12 (54.5)	0.076
Residual curvature	6 (42.8)	6 (27.3)	0.720
Clavien–Dindo			
- Grade 0	2 (14.2)	1 (4.4)	
- Grade 1	0 (0)	0 (0)	
- Grade 2	5 (35.8)	6 (27.2)	
- Grade 3	5 (35.8)	5 (22.9)	
- Grade 4	0 (0)	0 (0)	
Number of complications per patient	2.6 (2–3)	2.5 (1–4)	0.820
Functional outcomes			
Flow index (FI)	0.6715 ± 0.2419	0.8212 ± 0.2923	
- 1 = Tower > 1.253	0 (0)	2 (9.7)	0.060
- 2 = Bells > 0.659 < 1.253	8 (57.2)	11 (50.0)	
- 3 = Plateaus < 0.659	6 (42.8)	9 (40.3)	
IPSS score	7.69 ± 3.4	5.48 ± 3.0	
- 0= Mild (0–7)	6 (42.8)	16 (72.7)	0.050
- 1= Moderate (8–19)	7 (50.0)	5 (22.9)	
- 2= Severe (20–35)	0 (0)	0 (0)	
PPPS (0–12)	7.57 ± 1.95	8.27 ± 2.67	0.539
SIGHT (11–45)	25.71 ± 2.5	26.45 ± 2.7	0.794
HOSE (5–16)	11.74 ± 1.24	11.66 ± 1.22	0.257
HOPE (5–50)	43,36 ± 3.39	42.32 ± 6.78	0.599
Sexual Outcomes			
Erectile dysfunction	5 sexually active	13 sexually active	0.681
(IIEF-15 Score)			
- No (score: 26–30)	3 (60)	9 (72.7)	
- Mild (score: 17–25)	1 (20)	3 (23.1)	
- Moderate (score: 11–16)	1 (20)	1 (7.7)	
- Severe (score: 0–10)	0 (0)	0 (0)	
First sexual intercourse (years)	18.0 ± 2.5	18.1 ± 3.7	0.939
Gender identity	4.55 ± 0.1	4.54 ± 0.11	0.439
Sexual orientation	39.0 ± 13	51.1 ±28.8	0.634
BDI-II score	9.50 ± 6.1	3.14 ± 2.6	0.001
MAST	86.0 ± 10.4	86.0 ± 10.4	
Low tendency	2 (14.2)	2 (9.7)	0.634
Hight tendency	12 (85.8)	20 (80.3)	

## Data Availability

The original contributions presented in this study are included in the article. Further inquiries can be directed to the corresponding authors.

## Abbreviations

F.R.E.S.H.	Follow-up and Reconstruction in Ex-hypospadias Surgery for Hypospadias
STAI-Y1-Y2	State Anxiety Inventory
BDI-II	Beck Depression Inventory-II
MAST	Multi-Attitude Suicide Tendency Scale
IIEF-15	International Index of Erectile Function (15-item version)
PPPS	Pediatric Penile Perception Score
SIGHT	Satisfaction in Genital Hypospadias Treatment Questionnaire
HOSE	Hypospadias Objective Scoring Evaluation
HOPE	Hypospadias Objective Penile Evaluation
IPSS	International Prostate Symptom Score
PVR	Post-Void Residual Volume
VV	Voided Volume
Qmax	Maximum Urinary Flow Rate
Qave	Average Urinary Flow Rate
FI	Flow Index
KSOG	Klein Sexual Orientation Grid
IDYQ-AA	the Gender Identity/Gender Dysphoria Questionnaire for Adolescents and Adults

## Appendix A

### Appendix A.1. Study Protocol

The **F.R.E.S.H. study** involved the recruitment of all adult males (>18 years) who had undergone urethroplasty for hypospadias during childhood, performed by a single experienced paediatric urologist (GM). A research protocol was developed beforehand, and the study received approval from the Ethics Committee of the institution where it was conducted. The study commenced on 1 July 2021, with patient enrolment concluding on 1 September 2024.

All consecutive male patients who met the following criteria were included: being 18 years or older at the time of enrolment, having undergone urethroplasty before the age of 18, and having a confirmed diagnosis of hypospadias at birth. Signed informed consent to participate and allow data processing was required for inclusion. Exclusion criteria were established to maintain the study’s focus: female patients, individuals younger than 18 years at enrolment or older than 18 years at the time of surgery, and those who declined to provide informed consent were excluded. Patients who agreed to participate but failed to complete either the questionnaires or the clinical evaluation were also excluded from the final analysis.

Selection bias risks were acknowledged as inherent to retrospective studies, particularly challenges in identifying eligible participants due to incomplete paper or electronic records, obtaining accurate contact information such as phone numbers or email addresses, and motivating patients to participate. Patients in good health might be less inclined to respond compared to those dissatisfied with their outcomes. To minimise selection bias, clear inclusion and exclusion criteria were defined from the study outset. Additionally, data from patients who declined to participate were also collected, allowing for comparison between those who participated and those who did not. This approach helped to reduce the risk of bias associated with the hypothesis that patients experiencing problems are more likely to participate than those in good health. Efforts were made to accommodate participants with flexible dates for medical and psychological consultations, fostering motivation to participate.

Data were collected retrospectively from patient medical records (paper or electronic) or via patient interviews. Patients, or their parents/caregivers, were initially contacted by telephone, informed about the study, and asked to provide an email address for participation. Those who agreed received an e-mail containing a link to the questionnaires, which were to be completed independently after reviewing the study’s information and consenting to data processing. A brief telephone interview was conducted to collect additional variables. Participants were also asked to submit relevant medical documentation by email or bring it to their clinical examination.

Once the online questionnaires were completed, patients were scheduled for a urological evaluation, which included reconstructing their urological history, physical inspection, and uroflowmetry.

### Appendix A.2. Demographic and Clinical Variables

Sociodemographic data collected for each participant included age at the time of surgery, age at the time of the visit, years of education, and education level distribution. Employment status, marital status, and parental status were also recorded. Information on habitual medication use was also collected.

Regarding clinical and surgical history, hypospadias phenotypes were documented for each participant. Data on penile curvature and the presence of associated anomalies, such as cryptorchidism, were included. Additional procedures performed during surgery were documented, along with any complications observed during follow-up.

### Appendix A.3. Penile Outcomes

Penile appearance and patient satisfaction on surgery results were assessed using the Pediatric Penile Perception Score (PPPS) and the Satisfaction in Genital Hypospadias Treatment (SIGHT) [10,11]. The Hypospadias Objective Scoring Evaluation (HOSE) was used to assess the aesthetic outcomes of the urethroplasty [12]. The final aesthetic result was ultimately evaluated by the urologist in charge of the visit using the Hypospadias Objective Penile Evaluation (HOPE) scale to be as objective as possible [13].

### Appendix A.4. Urological Functional Outcomes

To evaluate long-term urological function, patients underwent uroflowmetry testing and post-void residual volume (PVR) measurement via ultrasound. Key uroflowmetry parameters, such as voided volume (VV), maximum urinary flow rate (Qmax), and average urinary flow rate (Qave), were recorded. The flow index (FI) was employed to classify the uroflow curves [14]. The International Prostate Symptom Score (IPSS) was utilized as a validated test to support the objectification of urinary symptoms, even though it is not specific to this population [15].

### Appendix A.5. Sexual Orientation and Gender Identity

In the study, participants provided information on their declared sexual orientation and perceived gender. Additionally, they completed two validated questionnaires: the Klein Sexual Orientation Grid (KSOG) and the Gender Identity/Gender Dysphoria Questionnaire for Adolescents and Adults (GIDYQ-AA).

The KSOG was developed to assess various aspects of sexual orientation, including sexual behaviour, attraction, sexual fantasies, social preferences, emotional preferences, self-identification, and lifestyle. These factors are evaluated across three time dimensions: the past, the present, and the ideal.

The GIDYQ-AA is a 27-item self-report measure designed to assess various dimensions of gender identity and dysphoria. The GIDYQ-AA evaluates subjective experiences, physical aspects, social interactions, and socio-legal considerations related to gender dysphoria. The questionnaire has demonstrated strong discriminant validity, effectively distinguishing individuals with gender dysphoria from control groups [16,17].

### Appendix A.6. Sexual Function Assessments

Sexual function was evaluated using the International Index of Erectile Function (IIEF-15) [18].

In addition, the Male Sexual Quotient (MSQ) and the Male Sexual Health Questionnaire (MSHQ) were used to further assess aspects of sexual health and function. The MSQ is a self-reported tool focusing on the emotional, physical, and functional dimensions of male sexual health [39]. The MSHQ assesses erectile function, ejaculation satisfaction, and overall sexual satisfaction, making it a comprehensive measure for sexual well-being [40].

### Appendix A.7. Mental Health Assessment

Mental health assessment was performed using a range of validated questionnaires. Anxiety symptoms were evaluated through the State Anxiety Inventory (STAI), a self-report questionnaire that measures the presence and severity of current symptoms of anxiety (STAI-Y1) and a generalized propensity to be anxious (STAI-Y2) [9]. A STAI-Y1 score ≥ 40 is indicative of pathological anxiety [19]. Depression was evaluated using the Beck Depression Inventory-II (BDI-II), while suicidal tendencies were assessed with the Multi-Attitude Suicide Tendency Scale (MAST). A BDI-II total score of 0–13 is considered minimal, 14–19 is mild, 20–28 is moderate, and 29–63 is severe [20,21].

Additionally, mental health was assessed using the Symptom Checklist-90-Revised (SCL-90-R) and the Short Form Health Survey (SF-36). The SCL-90-R is a comprehensive tool that evaluates a range of psychological symptoms, including anxiety, depression, and somatisation [41]. The SF-36 is a widely used survey that measures general health, functioning, and quality of life, assessing both physical and mental well-being [42].

### Appendix A.8. Handling of Missing Data

To ensure the robustness of the dataset, all patients who did not complete either the questionnaires or the clinical evaluation were excluded from the study. To minimise loss to follow-up, participants were reminded multiple times via email and phone calls and offered assistance in resolving technical issues with the online platform. Clinical evaluations were scheduled at flexible times to accommodate participants’ needs and maximise retention.

### Appendix A.9. Statistical Analysis

Data were analysed using IBM SPSS Statistics Base (v.29.0). Descriptive analyses were performed. The two groups identified by the presence of anxiety disorders (STAI-Y1< or ≥40) were compared by unpaired sample *t* tests for continuous variables and χ^2^ tests (with odds ratio—OR—calculation where appropriate) for qualitative variables. Statistically significant variables in the univariate analyses were then inserted as independent factors in a binary logistic regression model with presence of anxiety disorder as the dependent variable. The reliability of the model was assessed by the Omnibus and Hosmer–Lemeshow tests. Finally, correlation analyses were performed between STAI-Y1 scores and continuous variables.
